# Systematic Review of AI-Assisted MRI in Prostate Cancer Diagnosis: Enhancing Accuracy Through Second Opinion Tools

**DOI:** 10.3390/diagnostics14222576

**Published:** 2024-11-15

**Authors:** Saeed Alqahtani

**Affiliations:** Radiological Sciences Department, College of Applied Medical Sciences, Najran University, Najran 61441, Saudi Arabia; salqahtani@nu.edu.sa

**Keywords:** prostate cancer, artificial intelligence, MRI, machine learning, diagnostic accuracy, deep learning, imaging interpretation

## Abstract

Background: Prostate cancer is a leading cause of cancer-related deaths in men worldwide, making accurate diagnosis critical for effective treatment. Recent advancements in artificial intelligence (AI) and machine learning (ML) have shown promise in improving the diagnostic accuracy of prostate cancer. Objectives: This systematic review aims to evaluate the effectiveness of AI-based tools in diagnosing prostate cancer using MRI, with a focus on accuracy, specificity, sensitivity, and clinical utility compared to conventional diagnostic methods. Methods: A comprehensive search was conducted across PubMed, Embase, Ovid MEDLINE, Web of Science, Cochrane Library, and Institute of Electrical and Electronics Engineers (IEEE) Xplore for studies published between 2019 and 2024. Inclusion criteria focused on full-text, English-language studies involving AI for Magnetic Resonance Imaging (MRI) -based prostate cancer diagnosis. Diagnostic performance metrics such as area under curve (AUC), sensitivity, and specificity were analyzed, with risk of bias assessed using the Quality Assessment of Diagnostic Accuracy Studies (QUADAS-2) tool. Results: Seven studies met the inclusion criteria, employing various AI techniques, including deep learning and machine learning. These studies reported improved diagnostic accuracy (with AUC scores of up to 97%) and moderate sensitivity, with performance varying based on training data quality and lesion characteristics like Prostate Imaging Reporting and Data System (PI-RADS) scores. Conclusions: AI has significant potential to enhance prostate cancer diagnosis, particularly when used for second opinions in MRI interpretations. While these results are promising, further validation in diverse populations and clinical settings is necessary to fully integrate AI into standard practice.

## 1. Introduction

Globally, prostate cancer stands as the most frequently diagnosed form of cancer and ranks sixth among the leading causes of cancer-related deaths in men. Although it poses a significant public health concern, prostate cancer is highly manageable when detected in its early stages [1]. Generally, it is diagnosed by findings on a prostate-specific antigen (PSA) test and digital rectal examination [2]. Screening with PSA has resulted in a decline of more than 50% in mortality from prostate cancer [3]. However, it has also led to overdiagnosis and excessive treatment of mild forms of prostate cancer [4]. Prostate cancer can be incidentally identified through histological examination of prostate tissue obtained during transurethral resection of the prostate (TURP) performed for benign prostatic hyperplasia (BPH). Transrectal, ultrasound-guided (TRUS) prostate biopsy also serves as a standard diagnostic method for prostate cancer [5]. However, TRUS biopsy and invasive procedures like TURP are associated with high false-negative rates as well as problems like infection [6]. Additionally, the introduction of multiparametric magnetic resonance imaging (mpMRI) has brought about considerable progress in the detection of prostate cancer [7]. MRI, along with ultrasound-guided biopsies, has also shown improvement in the detection of clinically significant prostate cancer [8,9]. Yet, despite MRI’s excellent sensitivity for this cancer, it is constrained by moderate inter-reader reproducibility and low specificity [10]. Recent advancements in artificial intelligence (AI) offer potential solutions to address these challenges. Studies have shown that AI exhibits potential in automating the evaluation of the classification and intensity of prostate cancer through image-based assessments, encompassing MRI scans and histopathological analysis [11,12]. AI is creating a huge surge in radiology, as it has the capacity to offer speed and precision along with a second opinion for medical diagnostics and imaging.

A second opinion is a common practice in healthcare and comprises seeking validation by using an additional assessment or interpretation from someone else, commonly another qualified healthcare professional [13]. It is obtained to ensure that the information given by the first healthcare provider is correct [13]. It is usually sought after receiving a preliminary diagnosis [14]. It also aids in ensuring that an accurate diagnosis is made, reducing diagnostic errors, and the likely course of action taken is appropriate. Sometimes, different experts offer varying approaches or perspectives to disease detection or its management. The second opinion facilitates the exploration of alternative diagnostic strategies or treatment options. The discontentment of the patient with the first diagnosis is thought to be a major motive to take a second opinion, as it allows recommendations for better treatment [15]. However, practically, sometimes arranging a second opinion can be challenging for patients. At times, the initial healthcare provider may feel offended or reluctant to share information when approached for a second opinion and it can be unaffordable and inaccessible [16]. On the other hand, artificial intelligence has the capability to analyze patient data, and provide secondary opinions on disease detection and treatment plans around the clock. AI has also demonstrated an ability to assist care providers in the interpretation of radiological images [17]. In fact, nowadays, the utilization of AI in interpreting images, as well as in decision-making, is gaining noteworthy popularity. Currently, AI uses both deep learning and machine learning approaches for detecting prostate cancer from MRI results. Numerous studies have presented encouraging findings regarding the detection or description of prostate cancer from MRI results using AI, indicating that AI can offer improved decision-making or second opinions for patients with prostate cancer [18,19,20].

Given AI’s rapid evolution, this study aimed to offer updated insights into the diagnostic support provided by artificial intelligence in classifying and interpreting MRI images of prostate cancer. A systematic review was conducted to explore the effectiveness of AI in facilitating second opinions for MRI image readings in the context of prostate cancer diagnosis. A systemic review was performed because it assisted in extracting precise and high-quality literature on prostate cancer and AI from the vast volume of literature available. It also facilitated a full, concise, and accurate understanding of the relevant articles [21]. It offered clear insights into trends, gaps, and the overall usefulness of novel and different AI-based technologies for prostate cancer diagnosis using MRI data. Insights gained from reviews can enhance patient care when effectively applied in clinical practice, policy development, and administrative decision-making [22]. For researchers, it decreased the chances of bias and enhanced the validity and reliability of their results [21]. Thus, we performed a systematic review with three major objectives: (1) to evaluate the AI-assisted diagnosis of prostate cancer via MRI images; (2) to determine the extent to which AI can offer a second opinion regarding prostate cancer diagnosis based on MRI images with accuracy and efficiency; and (3) to explore the potential of AI in improving treatment planning and patient outcomes by analyzing MRI data for prostate cancer staging and progression.

## 2. Materials and Methods

### 2.1. Literature Search

The systematic review was performed by following the guidelines of the preferred reporting items for systematic reviews and meta-analyses (PRISMA) guidelines [23]. PRISMA guidelines are a broadly acknowledged scientific framework for carrying out systematic reviews and include steps like identification, screening, eligibility, and inclusion. Databases such as PubMed, Embase, Ovid, MEDLINE, Web of Science, Cochrane Library, and IEEE Xplore were utilized for the extraction of the articles [23]. The articles were based on the literature available regarding artificial intelligence used as a second opinion for MRI image analysis, in order to improve prostate cancer diagnosis. Searches were planned to find all studies that assessed various artificial intelligence techniques as the second opinion detection method for prostate cancer. Only pertinent articles were included, while those that did not meet the standards were excluded. The review included only articles in English, published between 1 January 2019 and 1 April 2024.

### 2.2. Study Selection

The articles were regarded as eligible if they fulfilled all of the following inclusion criteria: articles with a study population of patients with diagnosed prostate cancer; articles that used AI to examine MRI images of the prostate gland to identify prostate cancer; full-text articles with a clear methodology and available findings. Articles with diverse study designs, like cross-sectional studies, prospective, cohort, or retrospective studies, were included.

Articles were identified by evaluating their important aspects using the PICO framework (population, intervention, comparison, and outcome) (Table 1). 

Case studies, editorials, animal studies, correspondence papers, posters, conference abstracts, and ongoing trials were excluded. Articles wherein only MRI was used to diagnose prostate cancer, without the use of AI (either ML or DL algorithms) were excluded. Articles not written in English, published before 2019 and lacking clear descriptions of their lesion annotation methodology in the mpMRI protocol were also excluded.

### 2.3. Search Strategy Employed to Identify Relevant Studies

The search strategy was made to extract pertinent articles from various databases. Appropriate keywords, along with Medical Subject Headings (MeSH) terms tailored to the topic, were utilized. Filters were applied for language, publication date, and study type to extract the relevant articles, along with the use of Boolean operators to refine the search. This optimized the search process to retrieve appropriate studies while reducing irrelevant results. A natural language model was employed to enhance the retrieval of pertinent articles. This is because this approach improves the search process by making it more user-friendly and intuitive. Also, it can understand and process the context and intent behind the queries [24].

Additionally, in order to find other potential articles, reference lists were manually explored, which served as a supplementary means. As a whole, the search strategy was developed to be methodical, reproducible, and comprehensive, so that all relevant articles were identified for inclusion in this systematic review.

### 2.4. Data Extraction and Quality Assessment

A systematic extraction of data was carried out via a standardized data extraction form so that consistency and accuracy could be maintained across included articles. The form consisted of important information such as sample size, study characteristics, AI techniques, MRI performed, outcomes measured, and necessary findings. Through this structured approach, a wide range of information from the articles was synthesized into a cohesive analysis. A table showing the extraction form detail is presented in Appendix A.

Moreover, the Quality Assessment of Diagnostic Accuracy Studies tool (QUADAS-2) was applied to perform a quality assessment of the included articles [25]. This tool facilitated the evaluation of the applicability of the studies included [25]. Likewise, QUADAS-2 also helped in analyzing the risk of bias in each of the included studies as “High”, Low”, or “Unclear”. It contains four major domains, namely patient selection, reference standard, index test, and the flow and timing [25,26]. Studies with a high level of bias or low quality were excluded [26].

### 2.5. Screening and Study Selection

Two authors independently screened the articles to select them, reviewing the abstracts, titles, and full texts without any conflicts. They also performed the quality assessment of the studies.

## 3. Results

**Figure 1:**
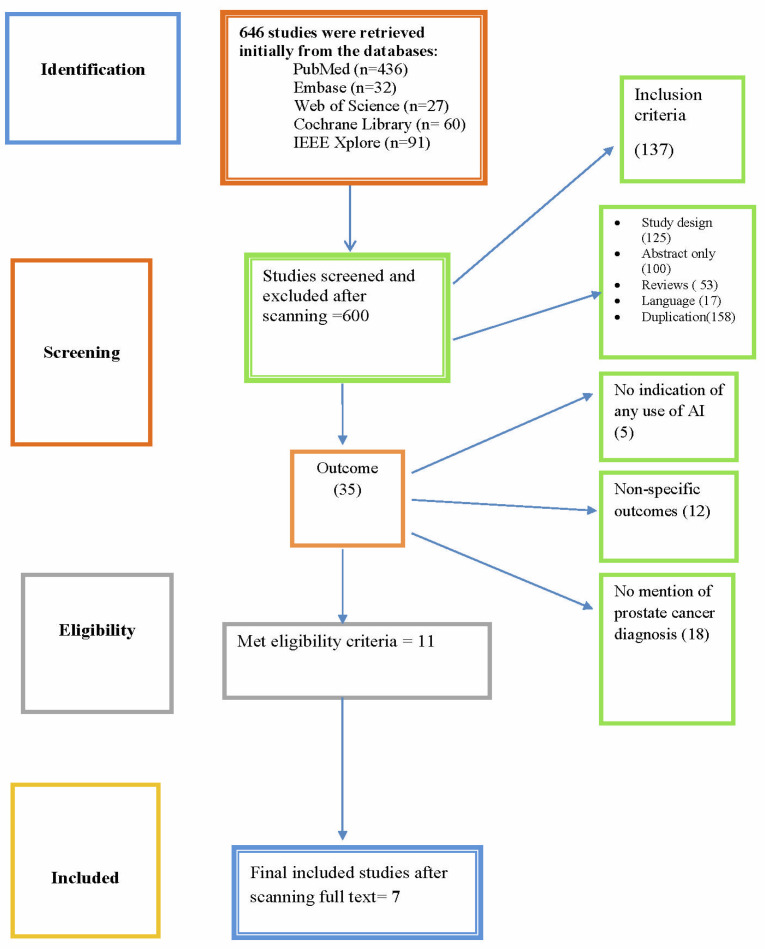
Flowchart for search results.

The search generated 646 studies, out of which 153 were only abstracts and reviews. They were excluded, and full-length articles were added. Seventeen studies were in a language other than English; these were also excluded. Following screening for a pertinent topic, eleven studies were selected. The RefWorks tool was used to exclude duplicate studies. Thirty-five full-length articles were also excluded, since they did not provide an apt outcome. Finally, seven studies were included in this review that accurately met the criteria. The PRISMA flow diagram is shown below in Figure 1.

### 3.1. Characteristics of Included Studies

Of the seven included studies, only one study, by Ström et al., was prospective; all others were retrospective study designs. They all used different types of AI algorithms; DL was used in four studies, ML was used in two studies, and CNN was used in three studies [27]. A study by Lee et al. used two models, i.e., ML and DL. Overall, deep learning was the most frequently employed AI technique in the included studies (Table 2) [28]. Four studies used mainly AI models that were developed based on radiological parameters. The remaining studies also incorporated clinical parameters like biopsies and Gleason score.

### 3.2. Study Outcomes

The included studies focused not only on detecting prostate cancer but also on its localization, scoring, and differentiation from benign disease. Lee et al., in their retrospective study, investigated the performance of both types of AI techniques [28]. They used image-based deep learning and texture-based machine learning for the diagnosis of prostate cancer in the transitional zone of the prostate. They evaluated both techniques in the settings of benign prostatic hyperplasia (BPH) and compared it with MRI and pathologically confirmed lesions [28].

Hosseinzadeh et al. demonstrated tumor localization using a deep learning model; in particular, they showed its usefulness for identifying Gleason lesions with a score above six [30]. Khosravi et al. developed and evaluated deep learning methods for distinguishing benign from malignant cancer, as well as differentiating high-risk and low-risk prostate disease [33]. Likewise, Yu et al., Salman et al., and Ström et al. employed diverse types of deep learning methods on patient biopsies and obtained satisfactory outcomes in terms accurately diagnosing prostate cancer using MRI data [27,29,31]. Hectors et al. was the only included study that used a machine learning model to predict the presence of prostate cancer [32]. All these studies used different parameters, such as biopsies of patients, histopathology images, magnetic resonance imaging data, and prostate tissue biopsy images. The studies by Lee et al. and Hectors et al. were the only ones that included real patients. These two studies directly involved human subjects in their research, as opposed to relying solely on simulations, models, or retrospective analyses. This distinction is noteworthy since including actual patients allows for more robust and clinically relevant findings because it reflects real-world outcomes and patient experiences [28,34].

Lee et al. found that texture-based machine learning algorithms had an AUC of 0.854–0.861 with high specificity, while image-based deep learning achieved a sensitivity of 0.946 and an AUC of 0.802 [28]. Hosseinzadeh et al. observed a sensitivity of 87% for detecting PI-RADS lesions ≥ four and 85% for Gleason lesions > six, using their deep learning model [30]. Khosravi et al. achieved AUCs of 0.89 for distinguishing cancer from benign cases and 0.78 for differentiating between high-risk and low-risk prostate disease with their AI model [33]. Yu et al. reported that their AI-based system outperformed over 70% of ordinary readers in MRI evaluations of prostate cancer [29]. Salman et al. achieved 97% accuracy on a test set of similar images and 89% accuracy on diverse biopsy images using their CNN-based system [31]. Ström et al. attained an AUC of 0.997 for differentiating between benign and malignant biopsies using their deep neural network model [27]. Hectors et al. recorded an AUC of 0.76 when using their random forest classifier to identify prostate cancer in PI-RADS 3 lesions [27]. 

### 3.3. Quality Assessment of Included Studies

The quality assessment of diagnostic accuracy studies (QUADAS)-2 quality was employed to assess the quality of the included studies, as shown in Table 3.

The methodological quality of the included studies varied. Most studies applied retrospective designs; this design can introduce potential biases and, therefore, limit the generalizability of the findings. For instance, Hosseinzadeh et al. and Khosravi et al. provided high sensitivity and specificity, but their studies’ retrospective natures may have affected the robustness of their results in real-world clinical settings [30,33]. The inclusion of prospective studies like Ström et al. offers a higher level of evidence, owing to that study’s reduced risk of bias and better handling of data [32].

## 4. Discussion

This systematic review meticulously analyzes the capability of artificial intelligence in detecting prostate cancer. The included studies were published within the last five years, demonstrating the rising awareness of AI’s use in oncology diagnosis. This systematic review also revealed the fairly high accuracy (with AUC scores of up to 97%) and moderate sensitivity of AI in the diagnosis of prostate cancer. The studies showed that AI-based diagnostic models have considerable potential in improving diagnostic accuracy for prostate cancer. For example, Lee et al. reported high specificity with texture-based machine learning models and excellent sensitivity with image-based deep learning [28]. This underlines the potential of combining different AI approaches to optimize diagnostic performance. Yu et al. showed that their AI-based system significantly outperformed ordinary readers, which highlights the potential of AI to enhance diagnostic consistency and accuracy [29]. Similarly, Salman et al. achieved high accuracy with a CNN-based system for classifying biopsy images, reflecting the effectiveness of AI in analyzing complex image data [31].

The emergence of increasingly powerful technology has led to the development of several AI models specifically designed for medical diagnosis. Research studies, both completed and ongoing, have demonstrated the effectiveness of AI in interpreting medical imaging and providing accurate diagnoses. There are possibilities of false positive rates due to conventional methods used to detect cancer. This can cause patients to undergo unnecessary invasive procedures like biopsies and treatments that lead to stress and more complications [35]. Not only do patients suffer from these issues, but false positives also burden healthcare systems with additional costs and resource allocation. Likewise, MRI has certain technological limitations, such as clarity and resolution problems, that influence diagnostic accuracy [36]. According to He et al., there is a need for a high level of proficiency, skill, and advanced tools to effectively interpret multiparametric MRI data [37].

In the face of such challenges, AI-based techniques can be used as second opinions to likely improve diagnostic accuracy. They can recognize subtle patterns and features of tumors that may be missed by human radiologists, improving early detection rates [38]. Pepe et al. demonstrated the relevance of obtaining a second opinion in men with equivocal PI-RADS 3 lesions [39]. They conducted an analysis of 950 cases obtained from both reference and affiliated radiological centers. The study found a significant difference in the diagnosis rates of clinically significant prostate cancer (csPCa) between these centers. Of the lesions diagnosed in the reference center, 26.7% were classified as csPCa, compared to only 16.6% in the affiliated centers [39]. Additionally, among lesions diagnosed by affiliated centers, 35.7% were downgraded and 15% were upgraded when reviewed by experienced radiologists from the reference center [39]. This underlines the necessity for a second opinion to optimize diagnostic accuracy and management. Similarly, using AI as a second opinion can address challenges such as limited access to specialized healthcare, particularly in low- and middle-income countries (LMICs) [40]. It can not only improve diagnostic accuracy but also facilitate timely interventions for patients. Additionally, integrating AI technologies into screening initiatives can help healthcare systems in LMICs to overcome resource constraints, allowing more individuals to benefit from effective diagnostic tools, thereby reducing healthcare disparities [40]. This systematic review also demonstrated the effectiveness of various AI algorithms in offering second opinions for detecting prostate cancer. This combination of AI advancements in pathology and the evidence supporting the need for second opinions can notably enhance patient management strategies in the diagnosis of clinically significant prostate cancer [39]. This also confirms the potential of AI in advancing digital pathology across a wide range of diseases.

These results are consistent with a study by Talaat, El-Sappagh, Alnowaiser, and Hassan, who proposed a model for the automatic detection of prostate cancer, called the prostate cancer detection model (PCDM), based on Region-Based Convolutional Neural Network (R-CNN), a deep learning (DL) algorithm [41]. The model was able to process a large dataset of medical images, achieving an accuracy of 95.24%, a precision of 97.56%, a specificity of 97.09%, and a sensitivity of 97.40% [41]. The model offered better detection without drastically increasing the computational complexity or the need for unnecessary biopsies.

Additionally, diagnoses of prostate cancer from MRI data were also reviewed, and they were found to be functional in accurately detecting the cancer. This is consistent with a study by Jiang et al., in which an AI-based system for detecting prostate cancer was developed and evaluated using MRI [42]. They studied its results against 24 radiologists on more than 200 patients and found that it exceeded the clinicians in accurately detecting the prostate cancer using MRI [38]. Likewise, Schelb et al. examined AI performance using MRI in their retrospective study of 312 men [43]. They found it had a specificity of 31% and a sensitivity of 96% in detecting clinically significant prostate cancer; these data were comparable to the performance of the Prostate Imaging Reporting and Data System [43]. Cao et al. also proposed FocalNet, which is a novel multi-class convolutional neural network, designed for prostate cancer detection and Gleason score prediction using multi-parametric MRI data [44]. They evaluated it using a dataset of 417 patients and found out that it had high sensitivity for detecting index lesions (89.7%) and clinically significant lesions (87.9%), with one false positive per patient [44]. For Gleason score classification, FocalNet achieved an area under the curve of 0.81 for clinically significant prostate cancer (GS ≥ 3 + 4) and 0.79 for prostate cancer, with a Gleason score of ≥ 4 + 3 [44]. Rouvière et al. investigated the efficacy of various AI-based MRI algorithms in the detection and characterization of prostate cancer [45]. The study found that these AI algorithms provided more robust results by significantly reducing false positive findings.

In addition to interpreting radiology imaging, there is an increasing demand for using artificial intelligence to study MRI-guided biopsies. This systematic review also showed that AI successfully detected the cancer from MRI-based data of prostate biopsies. This is in agreement with a study by da Silva et al., who evaluated Paige Prostate, an AI-based automated system designed for the detection of prostate cancer [46]. The study compared the effectiveness of Paige Prostate to the results obtained by pathologists using 600 biopsies from 100 patients to diagnose prostate cancer. Upon examination, Paige Prostate successfully diagnosed patients with prostate cancer with a specificity of 93% and a sensitivity of 99% [46]. It could precisely recognize certain parts of the prostate that were cancerous instead of an unreasonably high number of parts as doubtful. The results also showed that Paige Prostate diagnosed patients that three experienced histopathologists had previously failed to diagnose.

Nevertheless, while studies agree on the promising results of AI-based technology in accurately diagnosing prostate cancer, there are still more approaches required, as is clear when this technology is compared to the usual practices and techniques adopted by clinicians to detect prostate cancer. For example, Cuocolo et al. found that machine learning-based systems interpret prostate MRIs with good accuracy, yet they highlighted the need for the improved reporting of results as well as standardization in design [47]. AI models are only as effective as the data they are trained on. In case the dataset does not represent a wide-ranging patient population, biases can emerge. Similarly, a systematic review by Stanzione et al. reported a lack of features in prostate MRI radiomics that would support their introduction into clinical practice [48]. Also, while inconsistencies can arise between the initial assessment and the second opinion, clinicians need to initiate a collaborative review process in such cases [49]. This process may involve multidisciplinary discussions among urologists, radiologists, and pathologists to evaluate the findings comprehensively [49]. Moreover, further imaging studies or repeat biopsies may be performed to achieve a consensus on the diagnosis. Additionally, Prostate-Specific Membrane Antigen Positron Emission Tomography/Computed Tomography (PSMA PET/CT) can be used since it has emerged as a valuable imaging tool in the diagnosis of prostate cancer. It offers enhanced sensitivity and specificity compared to conventional imaging methods. This modality is particularly effective in detecting lesions that may be missed by traditional approaches and therefore it helps improve diagnostic accuracy [39]. Further research into this area is needed to bridge this gap.

The present systematic review attempted to investigate the effectiveness of AI-based MRI detection of prostate cancer so that it can be used as a second opinion. It is believed that AI could enhance the specificity of MRI interpretations, reduce the rate of false positives and minimize unnecessary interventions.

### Limitations

This study has several limitations. First, the selection of studies was restricted to those published in English, potentially excluding relevant research from non-English-speaking regions. Second, control data were used to trained AI models that might not be the cause of real-life scenarios. Third, the variability in datasets, AI algorithms, patients’ parameters, study designs, and sample sizes across the studies introduced heterogeneity, which could have affected the overall findings. The review therefore lacked external validation and generalizability. Moreover, this review offers a limited scope as it lacks information about AI’s ability to handle the distinct natures of different data types and disease pathologies. This may affect the broader applicability of the results. This study also largely overlooked the practical aspects of radiologists and oncologists applying AI technologies in real-world settings.

## 5. Conclusions

The systematic review showed different AI-based algorithms for prostate cancer detection and remained successful. This explains that AI has a substantial potential for improving diagnostic accuracy and patient outcomes. AI-based systems can help by providing timely detection and can be utilized for those whose biopsies are awaiting for full evaluation. They can assist medical professionals in improving the effectiveness and accuracy of clinical diagnoses and interventions, ultimately resulting in better patient outcomes.

Current research on prostate cancer predominantly centers on its detection and diagnosis. To maximize the potential of AI technology, future studies should investigate its applications in the treatment of prostate cancer and similar conditions. Similarly, they should attempt to validate AI models in diverse, multi-center settings, to ensure their generalizability.

Additionally, future research on AI algorithms for identifying prostate cancer should consider ethnicity and cultural variations. This will help guarantee that AI systems can be equitable and effectively implemented across diverse backgrounds and cultural communities, making them more accessible and inclusive.

## Figures and Tables

**Table 1:** The PICO criteria for this review.

Population	Adult Patients Diagnosed with Prostate Cancer or at Risk
Intervention	AI-based technologies for the detection and diagnosis of prostate cancer
Comparison	Traditional diagnostic approaches such as digital rectal examination (DRE), prostate-specific antigen (PSA) testing, transrectal ultrasound (TRUS), and prostate biopsy.
Outcome	Diagnostic accuracy, specificity, sensitivity, or improvements in overall clinical management.

**Table 2:** Summary of the results and characteristics of the included studies.

Author Name and Year	Objectives	Study Design	Types of Artificial Intelligence Model	Algorithm Performance	Conclusions
**Lee et al., 2023** [28]	To explore the performance of machine learning and deep-learning for identification of prostate cancer in the setting of benign prostatic hyperplasia (BPH).	A retrospective study.	Texture-based machine learning (support vector machine, logistic regression, and random forest) and image-based deep learning model (Convolutional Neural Networks).	Texture-based machine learning algorithms’ AUC is 0.854–0.861 with high specificity (0.710–0.775). The image-based deep learning demonstrated high sensitivity (0.946), AUC (0.802), and moderate specificity (0.643).	Both AI models can serve as an important tool for the diagnosis of prostate cancer.
**Yu et al., 2023** [29]	To develop, as well as authenticate, an AI-based system for the diagnosis of prostate cancer using MRI.	A retrospective study.	Deep learning (DL)-based AI-aided Prostate Imaging Reporting and Data System (PI-RADSAI).	Outperformed (45.5%).	The AI-based system outperformed more than 70% of ordinary readers in the MRI-based diagnosis of prostate cancer.
**Hosseinzadeh et al., 2022** [30]	To evaluate the performance of a deep learning (DL) model based on the Prostate Imaging Reporting and Data System (PI-RADS) algorithm, for the detection of prostate cancer.	A retrospective analysis.	Deep Learning- Computer-Aided Diagnosis (DL-CAD) model.	The sensitivity of DL-CAD was 87% when identifying PI-RADS lesions which were ≥4. The DL sensitivity was 85% for the detection of Gleason lesions which were >6.	DL can correctly detect and localize prostate cancer.
**Salman et al., 2022** [31]	To develop an AI-based system for detecting prostate cancer that can automatically identify key areas and accurately classify them on a biopsy image.	A retrospective study.	CNN architecture of deep learning.	The developed tool achieved 97% accuracy on a test set of 50 similar images and 89% accuracy on a test set of 137 different real prostate tissue biopsy images.	AI-based computer vision methods, like object detection algorithms, can develop highly accurate prostate cancer diagnosis tools.
**Hectors et al., 2021** [32]	To evaluate a machine learning model’s ability to identify prostate cancer in PI-RADS 3 lesions, specifically targeting pathological grade group ≥2.	Single-center retrospective study.	A machine learning model (random forest classifier).	The trained random forest classifier achieved a significant AUC of 0.76 for predicting prostate cancer.	The machine learning classifier showed good performance for the identification of prostate cancer in PI-RADS 3 lesions.
**Khosravi et al., 2021** [33]	To develop an AI-based model for the early identification of prostate cancer using magnetic resonance (MR) images.	A retrospective study.	Convolutional neural networks-based AI-aided biopsy.	The AI techniques achieved AUCs of 0.89 for distinguishing cancer from benign cases and 0.78 for differentiating high-risk from low-risk prostate disease.	The trained model combined biopsy report data with MR images, enhancing predictions beyond what magnetic resonance images alone can achieve.
**Ström et al., 2020** [27]	To create an AI system with clinically reliable accuracy for detecting, localizing, and grading prostate cancer.	A prospective study.	Deep neural network (DNN) models.	The AI achieved an AUC of 0.997 for differentiating between benign and malignant tumor biopsies.	A DNN-based AI system successfully differentiated between benign and cancerous biopsy cores.

**Table 3:** QUADAS-2 quality assessment.

Author (s) and Year	Patient Selection	Index Test	Reference Standard	Flow and Timing	Overall Quality
Lee et al., 2023 [28]	±	+	+	±	±
Yu et al., 2023 [29]	+	+	+	+	−
Hosseinzadeh et al., 2022 [30]	+	+	+	±	−
Salman et al., 2022 [31]	+	+	+	±	−
Hectors et al., 2021 [32]	±	+	+	±	±
Khosravi et al., 2021 [33]	+	+	+	±	−
Ström et al., 2020 [27]	+	+	+	+	−

+ = low risk, ± = moderate risk, − = high risk.

## Appendix A

**Table A1:** Extraction form details.

FirstAuthor (Surname)	Journal	Year
Study Design		
Sample Size		
Population Characteristics		
Intervention/Exposure		
Comparator		
Outcome (s)		
Effect Size/Results	Quality/Risk of Bias	Comments
